# Citric Acid-Assisted Electrokinetic Remediation of Arsenic and Metal-Rich Acidic Mine Pond Sediments

**DOI:** 10.3390/toxics13111000

**Published:** 2025-11-20

**Authors:** Oznur Karaca

**Affiliations:** Department of Geological Engineering, Canakkale Onsekiz Mart University, 17100 Canakkale, Türkiye; oznurkaraca@comu.edu.tr

**Keywords:** acidic mine sediment, heavy metal/metalloid, EKR, citric acid, environmental protection

## Abstract

Mining activities in the study area have led to the formation of irregular depressions where rainwater accumulates, creating acidic mine ponds. The water in these ponds becomes contaminated through contact with mine wastes and bottom sediments, leading to the dispersion of toxic metals and metalloids into the surrounding environment and food chain. This study investigates electrokinetic remediation (EKR) of highly contaminated acidic mine pond sediments and evaluates the role of citric acid (CA) as a biodegradable and environmentally friendly chelating agent. The sediment was highly acidic (pH 3.35) and contained elevated concentrations of Al, Fe, Mn, and As. Laboratory-scale EKR experiments were conducted for 27 days under a constant potential gradient of 1 V/cm, using 0.1 M CA as the electrolyte. The results obtained from this study were compared with those obtained using deionised water (DIW) as the electrolyte. The results demonstrated that CA significantly enhanced metal mobility, leading to higher removal efficiencies for Al (82.4%), As (51.1%), Mn (32.9%), and Fe (29.5%) compared to DIW. The pH near the cathode remained more balanced, and metal precipitation was minimised. Furthermore, total energy consumption decreased by about 53% (from 551 to 262 kWh/m^3^), indicating improved process efficiency. These results reveal that CA-assisted EKR can be an effective and sustainable method for the remediation of highly acidic mine pond sediments.

## 1. Introduction

While mining activities contribute significantly to economic development, improper mining also causes serious environmental problems. The mine waste generated by these activities often contains heavy metals and other toxic compounds, posing a significant threat to environmental and ecological systems [1,2]. High concentrations of certain metals and metalloids, such as aluminium (Al), iron (Fe), manganese (Mn), and arsenic (As), in soil can have toxic effects on plants and soil ecosystems. Acidic soils significantly limit crop production worldwide. The primary limiting factors in acidic soils are the presence of toxic levels of Al and Mn, as well as inadequate levels of phosphorus (P). Al^3+^, whose solubility increases in acidic soil conditions, inhibits cell division and elongation in plant roots, suppressing root growth, which in turn significantly reduces water and nutrient uptake [3,4]. Normal Fe concentrations in plants range from 0.03 to 0.3 g/kg. In general, concentrations above 0.5 g/kg are considered toxic [5]; however, this threshold may vary depending on the plant species, its physiological characteristics, and the surrounding growth conditions. An excess of iron in the soil often results in the appearance of brown spots on leaves, that progress to necrosis. Moreover, it interferes with photosynthesis, diminishes plant growth and chlorophyll concentration [6], and intensifies oxidative stress through elevated production of reactive oxygen species [7,8]. Arsenic originates naturally from volcanic activity and rock weathering, and anthropogenically from industrial operations such as mining, smelting, exploration, and manufacturing [9]. It is considered one of the world’s most hazardous chemicals [10], especially in its inorganic form, which was reported to be a carcinogen [11,12]. Long-term inorganic As intake from drinking water and food can lead to serious health problems such as skin disorders, skin cancers, kidney cancers, and reproductive disorders [13]. Toxic effects on both humans and plants are observed when arsenic concentrations exceed 55 mg kg^−1^ in soil and 50 μg L^−1^ in water [10].

Toxic levels of these elements not only threaten agricultural yields but also jeopardize long-term soil health by reducing soil microbial diversity [14]. The bioaccumulation of heavy metals in nature also impacts human health through the food chain [15,16,17,18]. Moreover, excessive metal accumulation in the soil can cause aquifer contamination, allowing pollutants to migrate over longer distances [19]. Therefore, effective treatment of mining waste and removal of toxic components is crucial for environmental sustainability.

Electrokinetic remediation (EKR) has emerged as an effective method for removing heavy metals and other contaminants from low-permeability environments, such as soil and sludge. This method utilises a low-voltage electric field applied directly to the waste matrix, enabling the movement of ions, thus facilitating the mobilisation and removal of contaminants [20]. The success of electrokinetic treatment depends not only on the applied electrical parameters but also on the chemical properties of the electrolyte solutions (chelates) used. Although extensive research has examined EKR for organic/inorganic contaminants in spiked soils containing silt and clay [21,22,23], studies involving naturally contaminated soils such as saline soils, mine tailings, or sediments [24,25,26,27,28] remain relatively limited.

Recently, the combined use of biological and electrokinetic (EK) processes has become a significant research focus for treating soils contaminated with organic and inorganic contaminants. These hybrid approaches integrate EK transport with microbial activity or biosurfactant production to enhance contaminant degradation and metal mobilisation in an environmentally sustainable manner [29,30]. These developments highlight the growing interest in integrating EK systems with green and biocompatible agents.

Various chelating solutions have been widely employed in EK systems to enhance the mobilisation of heavy metals. While deionised water (DIW) is frequently used as a reference electrolyte, its weak complexation capacity limits the removal of strongly bound metals [31]. On the other hand, organic acids, particularly environmentally friendly and biodegradable compounds such as citric acid (CA), act as highly effective chelating agents in EK treatment due to their capability to form stable complexes with metal ions [32]. CA, owing to its natural origin and low environmental impact, represents an advantageous alternative for sustainable remediation technologies, and also favours electro-osmotic flow [33,34]. Furthermore, previous studies conducted on both laboratory-spiked soils [35,36] and mine wastes/dredged sediments [27,37,38] showed that CA-assisted EKR enhances the mobility of metals and organic pollutants in various soils. Demir et al. [39] performed EKR on mine tailing soils contaminated with Pb, Cd, and Zn using 0.05 M ethylenediaminetetraacetic acid (EDTA) as a chelating agent. Their results indicate that EDTA did not significantly enhance EKR efficiency in highly contaminated mine tailings. Ortiz-Soto [40] examined the influence of pre-treating actual mining wastes with a strong acid, such as concentrated H_2_SO_4_, on the EK removal of Mn and Zn. Their findings indicate that acid pre-treatment enhanced metal mobility, achieving maximum net removal efficiencies of 31.88% for Mn and 17.95% for Zn.

In highly contaminated acidic mine sediments, the situation is more complex due to extremely low pH values, high Fe and sulphate contents, and strong metal-mineral associations. These conditions can significantly influence both the performance and mechanisms of EKR. Few studies have addressed such environments, and even fewer have evaluated the behaviour of arsenic together with major metals with biodegradable chelating agents. Therefore, the present study aims to investigate the efficiency of citric acid-assisted electrokinetic remediation for the treatment of real acidic mine pond sediments containing high concentrations of Al, Fe, Mn, and As. The objectives are to (i) evaluate the influence of CA on metal and metalloid mobility and removal efficiency, (ii) assess the effect of CA on pH control, especially in the cathode region and energy consumption, and (iii) compare the outcomes with deionised-water-based EKR. This research provides an understanding of the applicability and mechanisms of biodegradable chelating agents for the remediation of highly acidic, Fe-rich mine sediments, representing an important step toward sustainable management of contaminated mining sites.

## 2. Materials and Methods

**Figure 1:**
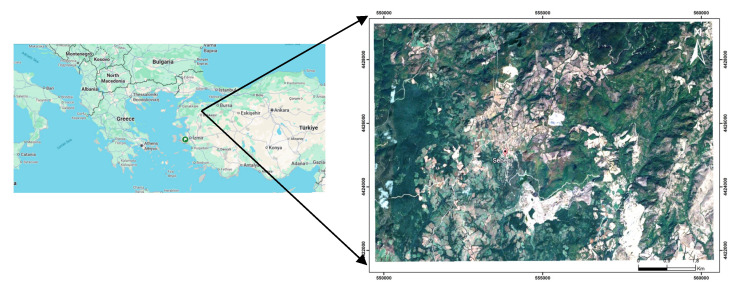
Location of the study area (GoogleMaps, 2025).

The region, including the study area (Figure 1), is rich in metallic and industrial minerals, such as quartz, feldspar, and kaolin, and energy raw materials, due to its diverse rock formations. A considerable amount of these resources is produced through open-pit and underground mining. In addition to metallic mines, the area also contains several coal mines, either still active or abandoned. The primary environmental issues associated with former mine sites in this region are the accumulation of mine tailings and the irregular topography of excavated areas. This uneven surface promotes the accumulation of rainwater and surface runoff, which in turn leads to the formation of acidic ponds. It was established in previous studies that mine waste and pond sediments are significant sources of toxic heavy metals, which contribute to the contamination of surrounding ecosystems [2,26,41]. For this study, pond sediments located in Sebepli (Balikesir, Türkiye) were collected using a shovel and stored in an airtight plastic bag. Sampling was conducted at three representative points, each from 20 to 30 cm in depth, and samples were homogenised to ensure uniformity.

### 2.1. Soil Preparation and Characterisation

Since characterisation is crucial in environmental risk assessments, pond sediments from the mine site were thoroughly evaluated for their physical and chemical properties. The soil sample collected from the field was first analysed to determine its moisture content (ASTM D2216) and organic matter content (dry basis) (ASTM D2974). Subsequently, sieve analysis was conducted to assess the grain size distribution, in accordance with ASTM D422. Atterberg limits were determined, indicating that the material is non-plastic in nature. Specific gravity and permeability were also determined by following the standard procedure (ASTM D854 and ASTM D2434, respectively). To measure pH and electrical conductivity (EC), a 1:10 soil-water suspension was prepared and analysed using a multi-parameter instrument (Thermo Scientific™ Orion 5 star, Beverly, USA) in accordance with ASTM D1293 and ASTM D1125, respectively. Metal analyses were performed by an accredited external laboratory, in accordance with EPA Methods 200.7 and 3051A using Inductively Coupled Plasma Optical Emission Spectrometry (ICP-OES) (Agilent 710, Santa Clara, CA, USA). Quality assurance and control (QA/QC) procedures, including calibration verification, blank measurements, and recovery tests, were conducted in accordance with the laboratory’s internal validation protocol. The recovery rates for all elements were within the acceptable range (85–115%), and detection limits were consistent with the laboratory method validation data.

### 2.2. Electrokinetic Test

**Figure 2:**
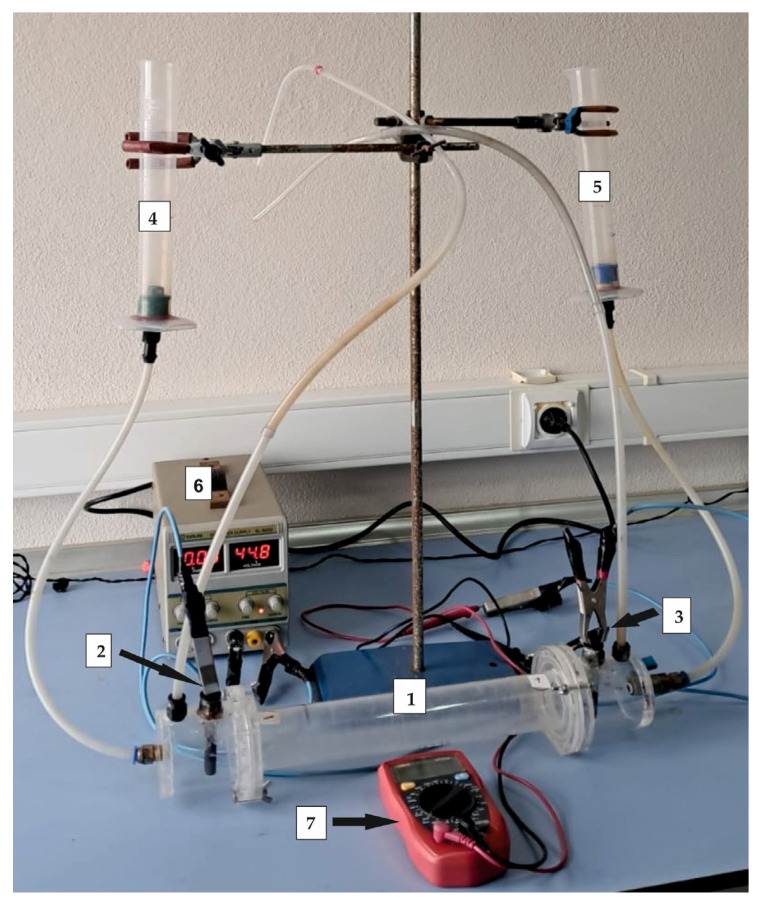
The electrokinetic test setup used in this study. (1) EK test cell, (2) anode, 3) cathode, (4) anode reservoir, (5) cathode reservoir, (6) power supply, (7) multimeter.

The EK setup included an EK test cell 22.3 cm in length and 4.0 cm in diameter, two electrode compartments, electrolyte reservoirs, and a power supply. The cell was constructed from Plexiglas, and graphite electrodes were installed in each electrode compartment, and were separated from the sample by 0.45 μm porous filter paper (Figure 2).

The soil sample was compacted into the electrokinetic cell in layers, taking care to prevent any voids between particles, and the reservoirs were then connected to the cell. A 0.1 molar CA solution was prepared as the electrolyte, as this concentration was reported to effectively enhance metal solubilisation and mobility during EKR while maintaining stable electrochemical conditions [36,42]. The electrolyte was filled into the reservoirs, and a constant voltage gradient of 1 V/cm was applied for the experiment. Throughout the test, the electric current and the water levels in both the anode and cathode reservoirs were regularly monitored and recorded. The experimental runs lasted for 27 days and were terminated upon a notable decline in current intensity. Upon completion of the test, the sample was taken from the cell and evenly divided into five sections. Following the determination of moisture content and physicochemical parameters of each section, arsenic and heavy metal concentrations were determined by ICP-OES.

To evaluate the effectiveness of the method, removal efficiency was calculated using the formula below:

$$R\%=\frac{C_{0}-C_{\mathrm{avg}}}{C_{0}}\times 100$$

$$C_{\mathrm{avg}}=\frac{1}{5}\sum_{i=1}^{5}C_{i}$$

here, C_o_ is the initial concentration, and C_i_ is the metal concentration in each segment (mg/kg). Total sediment masses used in the tests were 457 g (CA) and 359 g (DIW).

## 3. Results and Discussion

### 3.1. Characteristics and Environmental Impact of the Sediment

**Figure 3:**
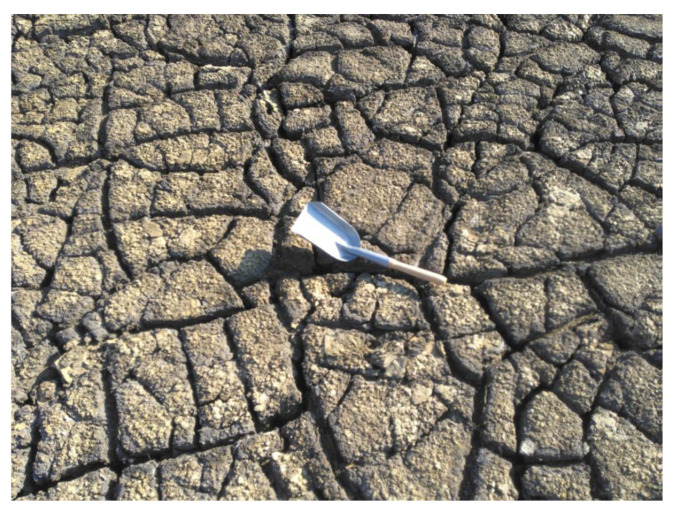
Lakebed view during sampling.

Two field visits were conducted in the study area. During the first visit, the mining site where coal was extracted was dry, allowing direct sampling from the lake bottom (Figure 3). During the second visit, in March, the mine pond was filled with water, part of which was draining into a nearby stream.

The physicochemical properties and leachability of the sediment collected from the lake bottom reveal significant environmental risks. The water content and organic matter content of the samples were determined to be 42% and 17%, respectively. Sediments were composed of 12% sand, 88% silt, and clay. Specific gravity and permeability of the sediment were 2.35 and 6.45 × 10^−6^ cm/s, respectively. The sample’s high acidity (pH = 3.35) and EC (2.427 mS/cm) indicate the presence of dissolved ions and potential contaminant load. High concentrations of aluminium (66,700 mg/kg), iron (56,800 mg/kg), manganese (751 mg/kg), and especially arsenic (1030 mg/kg) were detected in the sediment, posing a serious concern to the surrounding soil and water resources. Considering the initial values here, As far exceeds the limit values specified in the Regulation on the Regular Landfill of Waste for Türkiye [43]. According to the regulation, the inert limit value in the regulation is 0.05 mg/kg, the non-hazardous limit value is 0.2 mg/kg, and the hazardous limit value is 2.5 mg/kg.

In a previous study [26], column leaching experiments and sequential extraction analyses performed on sediment material revealed the mobility of these metals under environmental conditions. Column tests using deionised water demonstrated complete mobilisation of manganese, while 2.5% of aluminium and 1.5% of iron were converted to soluble forms. Despite its high total concentration, arsenic remained largely undissolved in water, and its mobility was found to be extremely low; this can be explained by the low water solubility of arsenic and its binding to persistent fractions within the sediment matrix.

To complement the leaching test results, the sequential extraction process was employed using the five-step procedure described by Tessier et al. [44]. This method enables the identification of metal speciation across five operationally defined fractions: exchangeable, carbonate-bound, Fe/Mn oxide-bound, organic-bound, and residual. According to the results, more than 95% of Al, Fe, Mn, and As are contained in the environmentally most stable residual fraction [26]. However, even these small mobile fractions can pose an environmental threat, especially under acidic conditions where metal mobility tends to increase.

The agreement between the sequential extraction and column leaching results reinforces the interpretation that Mn is the most environmentally mobile metal in the studied sediment, while As, Al, and Fe are more strongly retained within the sediment matrix. The low aqueous solubility of arsenic (particularly under acidic conditions) further explains its limited leachability, despite its high total concentration in the sediment [45]. This suggests that improved mobilisation techniques (e.g., chelating agents) may be necessary to facilitate removal during EKR.

In conclusion, both the high total concentration and partial mobility of toxic elements in the sediment indicate that the environmental impact of this material must be considered. Therefore, the evaluation of environmentally friendly methods, particularly electrokinetic treatment supported by biodegradable chelating solutions, is crucial for controlling this pollution.

### 3.2. EKR Application to Sediment

EK treatment experiments were conducted on sediment samples collected from a mining pond using a laboratory-scale setup that simulates one-dimensional contaminant transport. Two electrode chambers were placed at both ends, and in this study, 0.1 M CA solution was chosen as the chelator to enhance metal mobilisation, replacing deionised water used in previous experiments [26]. The CA-assisted experiments were performed 3 months after the DIW test using the same sediment batch and identical experimental setup. Sediment samples were stored under controlled laboratory conditions between tests to preserve their original characteristics.

**Figure 4:**
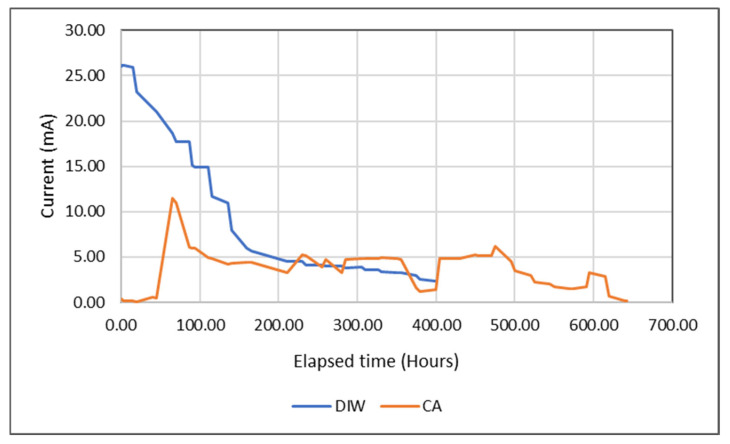
Electric current variation during EKR.

In this study, during the EK treatment process, a constant 1 VDC/cm (volt direct current/cm) potential gradient was applied to the sediment sample for 27 days (Figure 4).

The blue line in Figure 4 shows the current recorded during the EKR experiment in a previous study [26]. In this study, DIW was used as the electrolyte fluid, and a high current was obtained from the moment the electric field was applied to the soil. The increase continued for 17 h, reaching a peak value of 27 mA before a decrease in current values was observed. In the current study, CA was used as the electrolyte fluid, and the red profile was observed, as shown in the figure. Initially, low current values reached a peak value of 11.73 mA at hour 63, after which a decrease began, and it gradually decreased throughout the treatment process, dropping to 3.25 mA by day 9. In this experiment, a regular decrease was not observed as in the DIW experiment; on the contrary, fluctuations were observed. The overall trend was downward, and by the end of day 27, the current density had fallen to 0.21 mA (Figure 4). As a result, a decrease was observed in both experiments, which can be attributed to the gradual depletion of ions in the sediment. As the initially higher concentrations of soluble ions were transported to the electrode solutions, the EC of the matrix decreased, resulting in a corresponding decrease in current density. This ion transport primarily occurred through the electromigration mechanism, where anions migrate toward the anode and cations toward the cathode.

**Figure 5:**
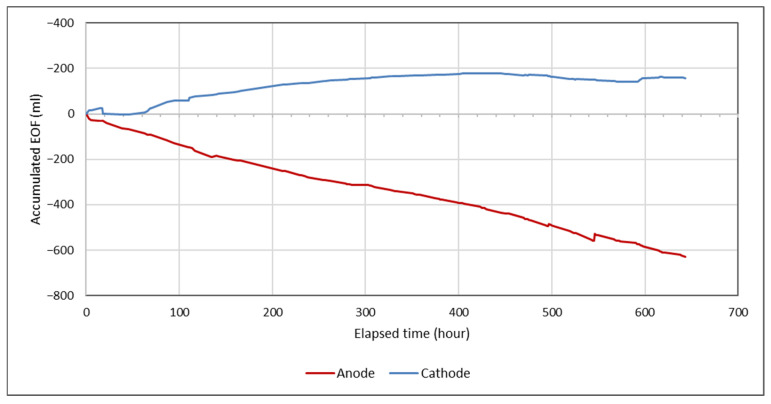
Accumulated EOF in the electrokinetic test with the sediment specimen.

In addition, electroosmotic flow (EOF) was observed during the electrokinetic remediation process in the system. EOF represents the volume of pore water transported through the sediment from the anode to the cathode under the applied electric field and the electroosmosis-induced flow rate was calculated by dividing the volume of water collected at the cathode reservoir by the elapsed time. The accumulated EOF refers to the total volume of water transported during the entire operating period. The flow was measured periodically using a graduated cylinder connected to the anode and the cathode reservoirs. Starting from day 17, the electroosmosis-induced flow rate decreased by 1 mL/h at the anode, while it continued to increase by 0.5 mL/h at the cathode until approximately day 27 (Figure 5). This electroosmotic flow contributed to the treatment process, particularly by supporting the transport of cationic metal species towards the cathode.

**Figure 6:**
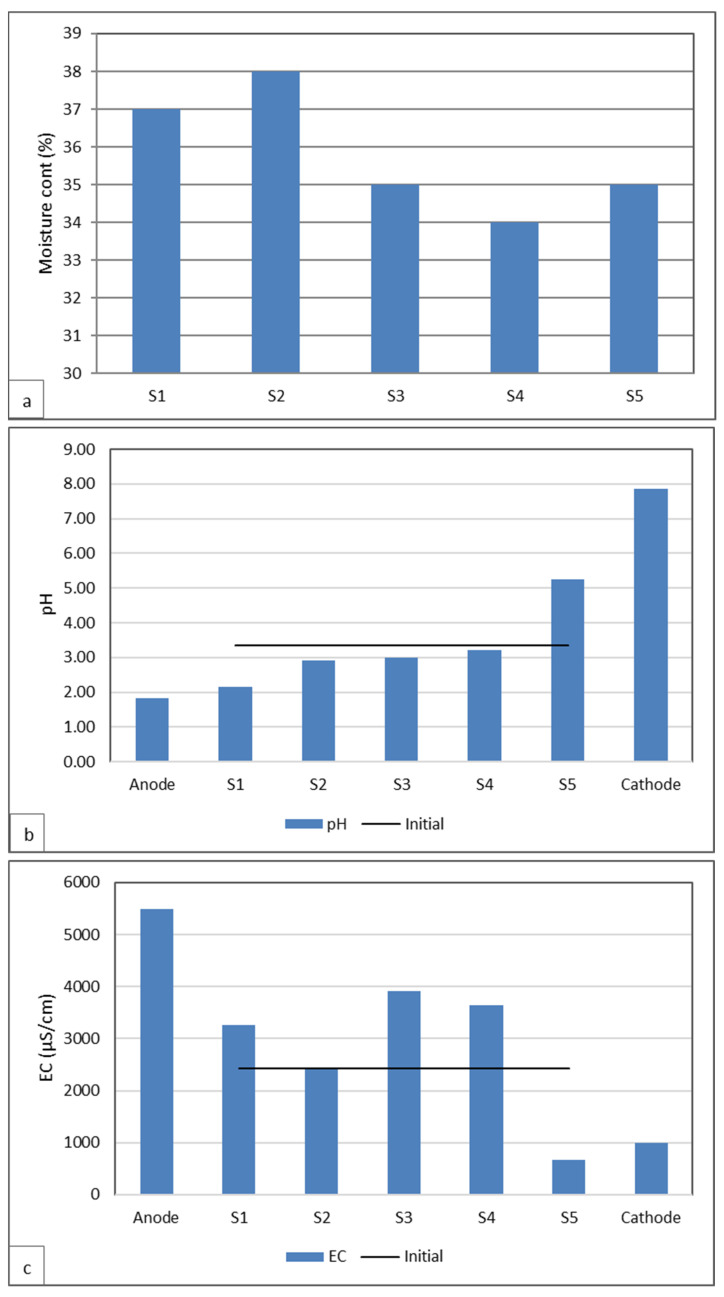
Variations in soil (**a**) moisture content, (**b**) pH, and (**c**) EC at the end of treatment.

**Figure 7:**
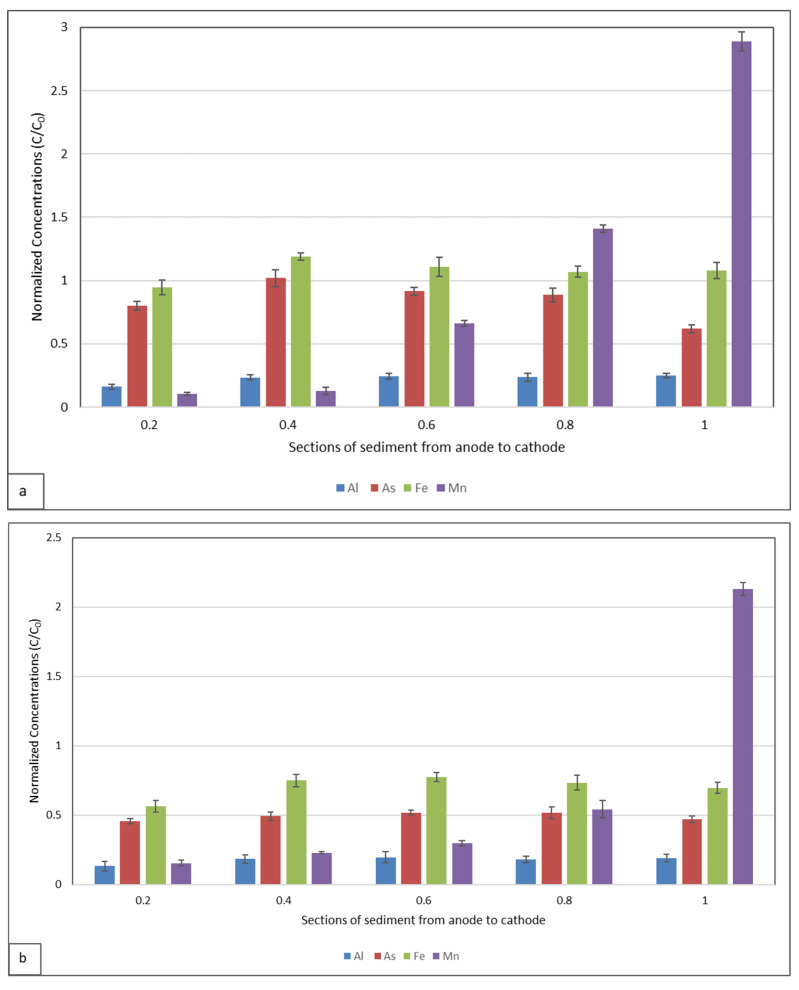
Metal/metalloid normalised concentrations in the sediment specimens after the EKR, (**a**) DIW results [26], (**b**) CA results. C and Co denote the final and initial metal concentrations in the soil, respectively.

At the end of the electrokinetic test, the sediment sample was removed from the cell and divided into five equal sections (S1–S5) from the anode to the cathode. Moisture content, pH, and EC were measured in each section, as well as in the electrode solutions (Figure 6). In addition, the metal concentrations in each section were determined (Figure 7). The EK test was started by saturating the soil sample to 35% in the laboratory. After EK treatment, the moisture content of the sediment sample remained very close to the initial value and was in the range of 34-38% in the soil sections. The acidification of the sample led to an increase in EC due to the high mobility of H^+^ ions. Low pH conditions increased the solubility of many metal species, leading to increased ionic concentrations and consequently increased EC values (Figure 6). The low EC values measured in the S5 region indicate effective removal of contaminant metals (Figure 7).

To analyse the transport of metals towards the electrodes, the solid phase metal concentrations (mg kg^−1^) measured in each sediment section (S1–S5) after electrokinetic treatment were compared with the corresponding initial total concentrations (C_0_) in the untreated sediment. The resulting ratios (C/C_0_) facilitate comparisons of the relative distribution and mobility of metals within the sediment profile, independent of their absolute concentrations. Metal concentration analyses were conducted in triplicate for each sediment section. Figure 7 shows the normalised concentrations (C/C_0_) of Al, As, Fe, and Mn for both the DIW control (a) and the CA-assisted system (b). Concentration profiles observed in sections of the sediment extending from the anode (S1) to the cathode (S5) revealed that positively charged ions migrated toward the cathode under the influence of the electric field. The low pH values of the sample favoured the presence of metallic elements in cationic forms. Therefore, both electromigration and electroosmosis played roles in the transport of toxic elements from the solid phase. These results, together with the EOF profile shown in Figure 5, provide evidence of directional metal fluxes and confirm that the use of citric acid increases metal mobility and overall process efficiency compared to DIW.

An evaluation of the data in Figure 7a demonstrates that Al was effectively removed by electrokinetic methods, while As and Fe were partially removed. The presence of Fe limited electrokinetic removal due to the mineral’s crystalline structure. Mn exhibited a distinctly different behaviour compared to the other elements. As seen in Figure 7, a distinct trend was observed in the transport and mobility of Mn from the anode to the cathode. Under acidic conditions, Mn dissolved in the S1 region and was transported to the cathode by electromigration. In the S4 and S5 regions, where high pH values prevail, Mn precipitated (Figure 7a).

In the study by Karaca et al. [26] (Figure 7a), the findings confirmed the effectiveness of the electrokinetic process in removing Al. The removal of iron and manganese from the sediment was not achieved. Instead, both metals were mobilised within the sediment matrix, migrating from the anode toward the cathode under the applied electric field. Mn exhibited a distinct migration pattern. As clearly illustrated in Figure 7, there is a pronounced trend indicating the mobilisation and transport of Mn from the anode to the cathode. Arsenic was only partially mobilised, suggesting that facilitating agents are required for its efficient removal. In contaminated environments, arsenic is mainly present as As(III) and As(V), with the latter being more mobile under alkaline conditions and more likely to migrate toward the anode due to its negative charge [46,47]. The strong affinity of arsenic for iron and aluminium oxides can limit its mobility, but the use of citric acid as a chelating agent can facilitate desorption by complexing Fe(III), thereby releasing previously immobilised arsenic [48,49,50]. Nevertheless, citric acid degradation under an electric field, particularly in zones with extreme pH, may reduce its effectiveness as a mobilising agent, affecting the overall efficiency [51]. While previous studies reported that As(V) species are more mobile under alkaline and electrokinetic conditions, our findings show that the speciation of arsenic remained largely unchanged with either water or citric acid treatment, indicating that As(III) predominates and remains bound to soil particles. This observation is consistent with Karaca et al. [26], who emphasised that effective remediation depends on longer operation times, cathode neutralisation, and the use of chelating agents. As shown in Figure 7b, citric acid application contributed to greater overall metal removal. Supporting these findings, measurable metal concentrations were also detected in both electrode chambers, particularly in the cathode compartment, confirming active electromigration. The concentrations in the anode and cathode chambers were as follows (mg/L): Al (1.78/6.02), As (0.39/<0.010), Fe (18.9/8.2), and Mn (0.24/7.12). These results validate the metal transport and accumulation at the electrodes, consistent with migration trends within the sediment.

Moreover, the calculated removal efficiencies (R) (Table 1) confirm these results. Although Al was effectively removed in both experiments, the CA-assisted treatment achieved higher removal. The most significant improvements were observed for As, Fe, and Mn, highlighting the pronounced effectiveness of CA use. The application of 0.1 M citric acid in this study is also consistent with previous findings reporting that this concentration efficiently enhances metal mobility and maintains favourable electrochemical conditions during remediation [36,42].

### 3.3. Evaluation of Energy Expenditure in EKR

Evaluating energy consumption is a key parameter for assessing the overall efficiency and cost-effectiveness of the EKR process, particularly when chelating agents are used as electrolyte solutions. Energy expenditure was determined using the following equation:

$$E=\frac{1}{Vs}\int VI\left(t\right)dt$$

here, V is the voltage, I is the average current measured during the EK test at time t in hours, and Vs is the volume of soil.

In this study, energy consumption was calculated for experiments performed with both DIW and CA. In the experiment using DIW, the total electrical energy consumption was approximately 551 kWh/m^3^ (0.235 kWh/kg). In contrast, in the experiment using CA, this value was 262 kWh/m^3^ (0.111 kWh/kg). This result indicates that the use of citric acid resulted in an approximately 53% reduction in energy consumption. This reduction can be attributed to the chelating agent increasing the electrical conductivity of the system and thereby reducing internal resistance losses. This facilitates ion migration and reduces the total energy requirements of the system.

## 4. Conclusions

This study evaluated the effectiveness of electrokinetic remediation (EKR) for treating highly contaminated mine lake sediments and the impact of citric acid (CA) as a biodegradable chelating agent on the process. The physicochemical characterisation of the sediment revealed that Al, Fe, Mn, and especially As were present at high levels, exceeding hazardous waste regulation limits and posing significant environmental risks. EK experiments conducted using CA showed that the chelator increased metal mobility and improved treatment efficiency compared to experiments using DIW. Among the metals studied, Mn exhibited the highest mobility under the applied electric field. In contrast, As and Fe were partially transported due to their strong binding within the sediment matrix, but their removal was enhanced compared to DIW. The use of CA contributed to maintaining pH balance throughout the cell, facilitated the transport of metal ions toward the cathode, and increased overall removal efficiency. Furthermore, energy consumption analyses showed that CA-assisted EKR reduced total energy requirements by approximately 53% compared to DIW. These results demonstrate that CA-assisted EKR offers an environmentally friendly and cost-effective alternative for the treatment of metal-contaminated sediments.

In future studies, higher voltage gradients may be applied to achieve shorter treatment durations and higher removal efficiencies, without causing adverse effects on soil health. This approach could facilitate large-scale, cost-effective field applications. Furthermore, post-EKR soil rehabilitation could be achieved through phytocapping (phytoremediation), which represents an environmentally sustainable and effective restoration method, particularly suitable for mining sites.

## Figures and Tables

**Table 1:** Removal efficiencies (R): CA vs. DIW.

Metal	C_0_ (mg/kg)	R (%) (CA)	R (%) (DIW)
As	1030	51.13	9.63
Al	66,700	82.39	77.3
Fe	56,800	29.54	0.0
Mn	751	32.86	0.0

## Data Availability

The original contributions presented in this study are included in the article material. Further inquiries can be directed to the author.
